# But Still It Moves: Static Image Statistics Underlie How We See Motion

**DOI:** 10.1523/JNEUROSCI.2760-19.2020

**Published:** 2020-03-18

**Authors:** Reuben Rideaux, Andrew E. Welchman

**Affiliations:** Department of Psychology, Downing Street, University of Cambridge, CB2 3EB, United Kingdom

**Keywords:** Bayes, direction, motion perception, natural images, neural network, speed

## Abstract

Seeing movement promotes survival. It results from an uncertain interplay between evolution and experience, making it hard to isolate the drivers of computational architectures found in brains. Here we seek insight into motion perception using a neural network (MotionNet) trained on moving images to classify velocity. The network recapitulates key properties of motion direction and speed processing in biological brains, and we use it to derive, and test, understanding of motion (mis)perception at the computational, neural, and perceptual levels. We show that diverse motion characteristics are largely explained by the statistical structure of natural images, rather than motion per se. First, we show how neural and perceptual biases for particular motion directions can result from the orientation structure of natural images. Second, we demonstrate an interrelation between speed and direction preferences in (macaque) MT neurons that can be explained by image autocorrelation. Third, we show that natural image statistics mean that speed and image contrast are related quantities. Finally, using behavioral tests (humans, both sexes), we show that it is knowledge of the speed-contrast association that accounts for motion illusions, rather than the distribution of movements in the environment (the “slow world” prior) as premised by Bayesian accounts. Together, this provides an exposition of motion speed and direction estimation, and produces concrete predictions for future neurophysiological experiments. More broadly, we demonstrate the conceptual value of marrying artificial systems with biological characterization, moving beyond “black box” reproduction of an architecture to advance understanding of complex systems, such as the brain.

**SIGNIFICANCE STATEMENT** Using an artificial systems approach, we show that physiological properties of motion can result from natural image structure. In particular, we show that the anisotropic distribution of orientations in natural statistics is sufficient to explain the cardinal bias for motion direction. We show that inherent autocorrelation in natural images means that speed and direction are related quantities, which could shape the relationship between speed and direction tuning of MT neurons. Finally, we show that movement speed and image contrast are related in moving natural images, and that motion misperception can be explained by this speed-contrast association not a “slow world” prior.

## Introduction

Humans use sensory systems to extract meaning from the environment: for instance, whether food is worth eating; the fitness of a mate; or the risks associated with particular actions. The mapping between sensations (e.g., changing patterns of light) and their physical causes (e.g., a nearby object's movement) is shaped by evolution and experience. Yet, it is difficult to isolate the causes and drivers of functional architectures found in nervous systems as evolution often eludes empiricism.

**Figure 1.**
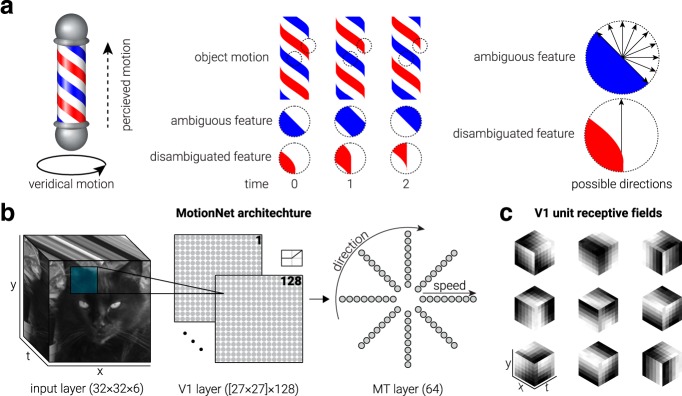
Barber pole illusion and MotionNet architecture. ***a***, When obliquely oriented stripes are rotated around a vertical axis, observers perceive upward motion. The local motion produced by features at the center of the pole is ambiguous (blue example), but features at the edge appear to move unambiguously up (red example). ***b***, MotionNet was initialized with an input layer, and convolutional and output layers representing V1 and MT, respectively. ***c***, Following training on motion sequences, kernels (V1 units) that were initialized as Gaussian noise formed 3D Gabors (the structure is partially obscured by the small kernel size but becomes clearer when the size is increased); nine examples, selected at random, are shown.

Perceptual illusions, where sensations appear decoupled from physical reality, question the relationship between environmental stimulation and our perceptual apparatus. In the barber pole illusion (Guilford, 1929; Wallach, 1935), for instance, viewers report that striped lines move upward in contrast to the objective rightward rotation of the pole (see Fig. 1*a*). This illusion reflects the ambiguity of local motion signals (Wallach, 1935); yet it remains a puzzle to understand the principles that dictate why our neural architecture is susceptible to this misperception in some situations but not in others. Moreover, we are challenged to explain a biological architecture molded by an uncertain combination of evolution and experience using isolated point estimates of network activity (e.g., electrophysiology) or gross population responses (e.g., fMRI).

Here we develop an artificial neural network trained as a toy system with which to interrogate the origin of functional architectures within the primate brain. Because we have complete control of the network's experience (i.e., its “visual diet”) and full access to the system, we can expose the processes that may underlie the perception and misperception of motion. This enables us to test key ideas about the influence of Bayesian priors and past experience. We assess how motion direction and speed are estimated to provide insight at the computational, neural, and perceptual levels. We use this new understanding to develop and test predictions from the artificial system against neurophysiological and psychophysical data to suggest the core biological computations of motion perception.

Using this analytical approach, we identify plausible drivers of biases in motion perception. In contrast to previous work, we show that the statistical structure of natural images per se, rather than the statistics of motion, can account for misperceptions of movement. First, we show that the overrepresentation of cardinal directions (i.e., up-down or left-right) in visual cortex is consistent with the structure of static images, not the statistics of environmental motion. Second, we reveal a statistical interdependency between movement speed and direction that can account for tuning properties of MT neurons. Third, we show (theoretically and empirically) that image contrast is a key “lurking variable” in motion estimation in ways not previously understood. Finally, we show that the brain appears not to have internalized the statistics of environmental motion (the “slow world” prior), but rather has learnt the statistics of image contrast dependent on the mutual information of natural images. In the process, we show that a neural network, optimized on one set of moving images, reproduces a host of biological neuron properties and perceptual behaviors, and supports concrete predictions for future experimental investigation.

## Materials and Methods

### 

#### 

##### Naturalistic motion sequences.

To train a neural network to classify the image velocity, we generated motion sequences using 200 photographs from the Berkeley Segmentation Dataset (https://www2.eecs.berkeley.edu/Research/Projects/CS/vision/bsds/). Images were grayscale indoor and outdoor scenes (converted from RGB using MATLAB's *rgb2grey* function, The MathWorks). Motion sequences (six frames) were produced by translating a 32 × 32 pixel cropped patch of the image (see Fig. 1*b*). Eight motion directions (four cardinal; four oblique) and eight linearly spaced speeds between 0.8 and 3.8 pixels/frame were used. Images were translated in polar coordinates; for example, an image moving at a speed of 1 pixel/frame in 0° (right) direction was translated by [*x* = 1, *y* = 0] per frame, whereas an image moving at the same speed in 45° direction was translated [*x* = 0.7071, *y* = 0.7071]. Image translation was performed in MATAB using Psychtoolbox version 3.0.11 subpixel rendering extensions (Brainard, 1997; Pelli, 1997) (http://psychtoolbox.org/). The speeds used to train the network were selected because they did not exceed the image dimensions (32 × 32 pixels), and all required subpixel rending; for example, if we had used a speed of 1 pixel/frame, subpixel rendering would have been required for cardinal, but not oblique, directions. We therefore ensured that all directions necessitated subpixel rendering to ensure that this did not provide a direction cue. We generated 64,000 motion sequences; 1000 for each of the (8) directions and (8) speeds. Image sequences were scaled so that pixel intensities were between −1 and 1, and randomly divided into training, validation, and test sets, as described in Training procedure.

##### MotionNet architecture.

All the networks described in the study were implemented in Python version 3.6.4 (https://python.org) using Tensorflow (www.tensorflow.org), a library for efficient optimization of mathematical expressions. We used a convolutional neural network that comprised an input layer, one convolutional-pooling layer, and an output logistic regression layer (see Fig. 1*b*). The input was convolved with a series of kernels to produce one output map per kernel (which we refer to as convolutional maps). The use of convolution means that each kernel is applied at all valid locations of the input space; valid locations refer to those in which the convolutional kernel does not exceed the input space. This significantly reduces the number of parameters that need to be learned (i.e., we do not parametrize all possible pairwise connections between layers) and allows the network to extract a given image feature at all different positions of the image.

Inputs were image patches (32 × 32 × 6 pixels; the last dimension indexing the motion frames). In the convolutional layer, inputs passed through 128 3D kernels (6 × 6 × 6 pixels), producing 128 2D output maps (27 × 27 pixels). This resulted in 36,992 units (128 maps of 27 × 27 pixels) forming 20,155,392 connections to the input layer (128; 27 × 27 × 6 × 6 × 6 pixels). Since mapping is convolutional, this required that 27,776 parameters were learned for this layer (128 filters of dimensions 6 × 6 × 6 plus 128 offset terms; we refer to these parameters as offset terms, rather than the standard “bias terms,” to avoid confusion with “perceptual bias”). We chose units with rectified linear activation functions to model neurophysiological data (Movshon et al., 1978). The activity, *a*, of unit *j* in the *k*^th^ convolutional map was given by the following:

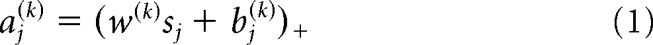
 where *w*^(*k*)^ is the 6 × 6 × 6 dimensional 3D kernel of the *k*^th^ convolutional map, *s*_j_ is the 6 × 6 × 6 motion sequence captured by the *j*^th^ unit, *b*_j_ is an offset term, and (…)_+_ indicates a half-wave rectification. Parameterizing the motion image frames separately, the activity *a*_*j*_^(*k*)^ can be alternatively written as follows:

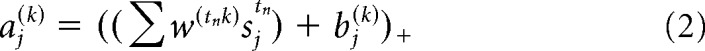
 where *w*^(*t_n_k*)^ represents the *k*^th^ kernels applied to motion image frames (i.e., receptive fields at times 1–6) and *s_j_^t_n_^* represents the input images captured by the receptive field of unit *j*.

Finally, a logistic regression layer (2,367,552 connections; 46,656 per feature map, resulting in 2,367,488 parameters, including the 64 offset terms) mapped the activities in the pooling layer to 64 output decision units. The vector of output activities *r* was obtained by mapping the vector of activities in the convolutional layer *a* via the weight matrix *W* and adding the offset terms *b*, followed by a *softmax* operation as follows:

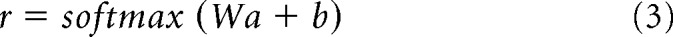
 where the predicted class was determined as the unit with highest activity.

##### Training procedure.

Motion sequences were randomly divided into training (70%, *n* = 44,800), validation (15%, *n* = 9600), and test (15%, *n* = 9600) sets. No sequences were simultaneously present in the training, validation, and test sets. To optimize MotionNet, only the training and validation sets were used. We initialized the weights of the convolutional layer as Gaussian noise (mean, 0; SD, 0.001). The weights in the logistic regression layer and all offset terms were initialized to 0.

MotionNet was trained using mini-batch gradient descent, with each batch comprising 100 randomly selected examples. For each batch, we computed the derivative of the categorical cross entropy loss of function with respect to parameters of the network via back-propagation, and adjusted the parameters for the next iteration accorded to the update rule as follows:

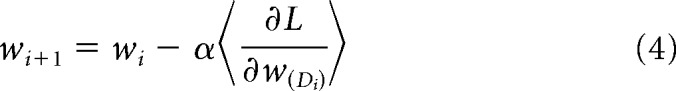
 where α is the learning rate and $\langle \frac{\partial L}{\partial w_{\left(D_{i}\right)}}\rangle$ is the average over the batch *D_i_* of the derivative of the loss of function with respect to the *w*, evaluated at *w_i_*. The learning rate α was constant and equal to 1.0e^−4^. After evaluating all the batches once (i.e., completing one epoch), we tested MotionNet using the validation image dataset. We repeated this for 2500 epochs.

##### Generalization of motion classification.

To test generalization by MotionNet, we used sequences from a realistic movie depicting the view from a car driving through a busy urban environment (“driving” dataset; https://lmb.informatik.uni-freiburg.de/resources/datasets/SceneFlowDatasets.en.html) (Mayer et al., 2015). The motion scenes comprise both self-motion generated optic flow and object motion, with ground truth provided. To extract sequences to test MotionNet, we segmented the movie (960 × 540 pixels, 800 frames) into smaller sections, equal to the input shape used by the network (32 × 32 pixels, 6 frames). We then removed motion sequences for which the average velocity was more or less than those used to train MotionNet, or for which the SD of the velocity exceeded 0.2 in terms of direction (degrees) or speed (pixels/frame). To increase the sample size, each motion sequence was rotated and repeated at each motion direction. This yielded 640 motion sequences (10 for each motion velocity used to train the network).

##### Generation of test stimuli.

A range of stimuli were used to test the response of the network after it had been trained on natural images. With the exception of sinewave and plaid stimuli, which were generated in Python using in-house scripts, all stimuli were generated using the Python toolbox Psychopy (Peirce, 2007) version 1.90.3 (http://www.psychopy.org). Except where stated otherwise, all sinewaves and plaids (90° component separation) had a spatial frequency of half the image size (16 pixels/cycle) and were presented at full contrast (−1 to 1 pixel values). In line with Stocker and Simoncelli (2006), contrast was defined as the ratio between the maximal intensity difference in the image sequence and the maximum intensity difference that could be input to the network (MotionNet) or displayed on the monitor (human observers).

##### Decoding continuous measures of direction and speed.

MotionNet was trained to perform discrete velocity classifications. To generate continuous estimates of speed and direction, we fit descriptive models to the activity of the MT units. For direction, we fit a von Mises distribution to the activity of units tuned to the same speed as that of the unit with maximum response according to the following:

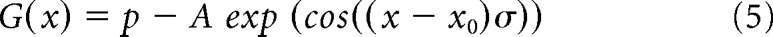
 where *G*(*x*) denotes the unit response at location *x*, *p* is a constant offset, and *A*, *x*_0_, and σ are the amplitude, location, and width of the peak, respectively.

Similarly, for the speed model, a Gaussian distribution was fit to the activity of units tuned to the same direction as that of the unit with maximum response, according to the following:

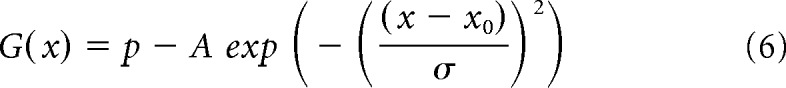
 where *G*(*x*) denotes the unit response at location *x*, *p* is a constant offset, and *A*, *x*_0_, and σ are the amplitude, location, and width of the peak, respectively.

For simulations in which velocity was computed from the MT unit population activity, stimuli were presented in each of the 8 (cardinal/oblique) directions, and the response of the MT units was aligned to a common direction (0°) and averaged.

##### Anisotropy in direction of motion responses.

**Figure 2.**
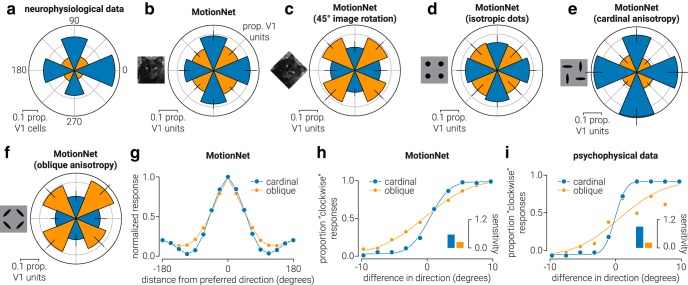
Anisotropy in direction of motion responses. ***a***, Data from Salinas et al. (2017) showing the proportion of spatiotemporal neurons in mouse V1 tuned to cardinal (blue) and oblique (orange) directions. ***b–f***, Same as in ***a***, but for V1 units of MotionNet when trained on (***b***) natural images, (***c***) natural images rotated by 45°, (***d***) random dots with isotropic orientation, or random dots with (***e***) cardinal or (***f***) oblique anisotropic orientation. ***g***, The average tuning functions of MotionNet MT units with cardinal or oblique preferred directions. ***h***, MotionNet's sensitivity (reciprocal of the just noticeable difference) to changes in direction around cardinal and oblique axes. ***i***, Replotted data from Matthews and Qian (1999) showing human sensitivity for direction discrimination around cardinal and oblique directions. ***b–f***, Error bars indicate SD.

To compare the properties of V1 units that emerged within the MotionNet with those of V1 neurons in biological systems, we extracted neurophysiological data of mouse V1 neurons selective for cardinal and oblique directions from Salinas et al. (2017, their Fig. 7D). That figure contains data presented separately for high (≥0.24 c/d) and low (<0.24 c/d) spatial frequency; we averaged those to capture the distribution of direction selectivity across all neurons (see Fig. 2*a*). To establish the direction tuning preferences of MotionNet V1 units, we tested the network with drifting sinewave gratings. The direction preference of each unit was determined as the stimulus movement direction that produced maximal activity (see Fig. 2*b*). Eight directions (four cardinal and four oblique), spatial frequencies (logarithmically space between 2 and 64 pixels/cycle), and temporal frequencies (logarithmically space between 2 and 64 cycles/frame) were tested, resulting in 512 (8 × 8 × 8) stimulus types. For each stimulus type, we computed the average activation of 32 gratings at evenly spaced starting phase positions between 0 and 360°. From these data, the spatial and temporal frequency selectivity of the V1 units was also derived and used in other analyses.

To test hypotheses about the cause of anisotropies in the distribution of V1 unit preferences, we used the mapping procedure on networks trained on both natural and artificial image sequences. A MotionNet (45° image rotation) network was trained on (the same) natural images, following a 45° clockwise rotation (see Fig. 2*c*). A MotionNet (isotropic dots) network was trained on artificial random dot motion stimuli comprising a variable number of dots (number between 1 and 10, randomly selected) of different size (8–16 pixel diameter, randomly selected) and contrast (pixel values between −1 and 1, randomly selected) on a mid-gray background (pixel value 0) (see Fig. 2*d*). Dots were randomly positioned within the image (32 × 32 pixels) ± the maximum distance that a dot could traverse over the sequence (6 frames × 3.8 pixels). During the motion sequence, dots translated across the image without wrapping around at the edges. For each of the eight directions and eight speeds, we generated 500 images on which to train the networks. MotionNet (cardinal anisotropy) and MotionNet (oblique anisotropy) networks were trained on similar image sequences, but the dots were distorted along cardinal or oblique axes by a factor of 4 (see Fig. 2*e*,*f*). This was achieved by reducing the width of the dots by half and increasing the height by a factor of 2, and then (randomly) rotating each dot to align with either the cardinal or oblique axes. To demonstrate and ensure consistency in training outcomes, we trained 10 networks on each set of image sequences. In Figure 2, we present the mean values, with error bars indicating SD.

To establish the direction tuning width of MT units (see Fig. 2*g*), we used random dot stimuli: tested directions, 0°-360° in 16 steps; speed, 0.8–3.8 pixels/frame in 8 steps. Dot motion stimuli consisted of 12 randomly positioned white dots (pixel value, 1; radius, 5 pixels) on a black background (pixel value, −1), which were allowed to overlap (with occlusion) and wrapped around the image when their position exceeded the edge. Responses were then aligned to the preferred direction and averaged across MT units tuned to either cardinal or oblique directions.

We measured the sensitivity of MotionNet to changes in direction around cardinal and oblique directions, and fit a descriptive model (von Mises distribution) to obtain a continuous output from the network to dot motion stimuli moving in a range of offsets (±20° from the target direction in 10 steps; speed, 3.8 pixels/frame). To generate “psychophysical performance” from MotionNet, each offset was repeated 10 times and the proportion of “clockwise” responses; that is, decoded (continuous) direction clockwise relative to 0, was plotted as a function of offset (see Fig. 2*h*). These data were then averaged for cardinal or oblique directions to produce one measure of psychophysical performance for cardinal directions and one for oblique. The MATLAB toolbox Psignifit (Fründ et al., 2011) (http://psignifit.sourceforge.net/) was used to fit psychometric functions. Measures of sensitivity were calculated as follows:

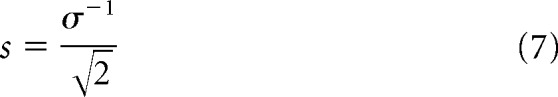
 where *s* denotes sensitivity and σ is the SD of the psychometric curve. For comparison with human data, we extracted the psychometric data points from Matthews and Qian (1999, their Fig. 1, top left) and used the same method used to calculate the psychometric functions and measures of sensitivity of MotionNet (see Fig. 2*i*).

##### Biological and artificial visual system responses to component and pattern motion.

**Figure 3.**
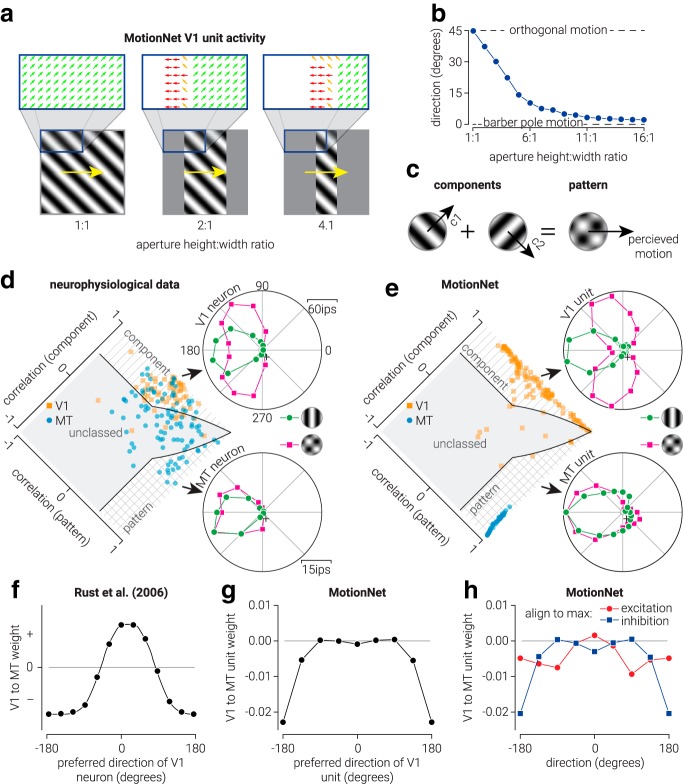
Biological and artificial visual system responses to component and pattern motion. ***a***, Illustrations of barber pole stimuli and the evoked responses in MotionNet. Arrow colors represent different directions. ***b***, MotionNet's estimate of the movement direction as a function of the aspect ratio of the stimulus. ***c***, Illustration of how two “component” sinewave gratings moving in different directions form a plaid “pattern,” which moves in a (different) third direction. ***d***, Data from Adelson and Movshon (1982) showing single-neuron responses in V1 (top) and MT (bottom) to a sinewave grating versus a plaid stimulus. The distribution plot shows the population of single-neuron responses, and whether they are classified as component-motion or pattern-motion selective. ***e***, Same as in ***d***, but for MotionNet V1 and MT units. ***f***, Illustration of the V1 to MT neuron readout weights proposed by Rust et al. (2006). ***g***, The average weights from MotionNet's V1 to MT units organized by preferred V1 direction. ***h***, Same as in ***g***, but organized such that the weights between V1 and MT units are all aligned to direction 0 at either their maximum or minimum weight.

To assess MotionNet's response to barber pole stimuli, we tested the network with obliquely oriented drifting sinewave gratings (speed, 0.8 pixels/frame) that were masked at the edges (pixel value, 0) to obtain 16 height to width ratios from 1:1 to 16:1 (see Fig. 3*a*). To obtain ratios that could not accurately be achieved by masking one pair of edges (e.g., the left and right edges), due to the limited number of pixels within the image, the other pair of edges (e.g., the top and bottom edges) were also masked in a manner that maximized the total number of pixels left unmasked while accurately achieving the desired ratio. This process was repeated for each of the four cardinal directions, and the response was aligned and averaged to a common direction before decoding. This was done to ensure equality of responses between units tuned to directions within cardinal/oblique direction groups (e.g., responses of units tuned to upward motion were similar to those tuned to downward motion). Finally, we decoded (continuous) direction from MT activity for stimuli as a function of height to width ratio (see Fig. 3*b*). To visualize V1 unit activity in response to the barber pole stimuli, we considered an array of units across the V1 layer that receive input from specific regions of the stimulus and labeled each point with the direction corresponding the maximally active V1 unit (see Fig. 3*a*). Only a portion of this activity is shown, as the stimulus and corresponding activity were symmetrical around the midline.

To compare the responses of MotionNet units to electrophysiological recordings from neurons in macaque V1/MT (Movshon et al., 1983, extracted and replotted neurophysiological data from their Figs. 11–13), we measured the activity of V1/MT units in response to sinewave gratings and plaids (135° separation) moving in 16 evenly spaced directions between 0° and 360° at its preferred speed (see Fig. 3*e*). The preferred speed of the V1 units was established as the spatial and temporal frequency to which they responded maximally, and the preferred speed of the MT units was the speed for which they were designed to classify. To ensure consistency with the empirical data, we used the same methods as Movshon et al. (1983), to measure the responses of V1 and MT units, with the only exception that we tested plaid stimuli with an angular separation of 90° (rather than 135°).

To classify each unit as component-selective (i.e., selective for the motion of the individual components comprising a plaid pattern), pattern-selective (i.e., selective for the motion of the plaid pattern), or unclassed (see Fig. 3*e*), we used the method described by Movshon et al. (1983). Briefly, we compared the unit responses with ideal “component” and “pattern” selectivity using goodness-of-fit statistics. As the component and pattern selectivity responses may be correlated, we used the partial correlation in the following form:

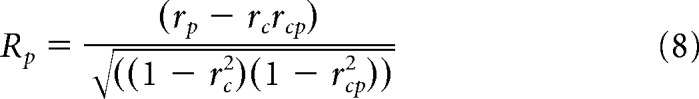
 where *R_p_* denotes the partial correlation for the pattern prediction, *r_p_* is the correlation of the data with the pattern prediction, *r_c_* is the correlation of the data with the component prediction, and *r_cp_* is the correlation between the two predictions. The partial correlation for the component prediction was calculated by exchanging *r_c_* for *r_p_* and vice versa. We labeled units as “component” if the component correlation coefficient significantly exceeded either 0 or the pattern correlation coefficient, whichever was larger. Similarly, we labeled units as “pattern” if the pattern correlation coefficient significantly exceeded either 0 or the component correlation coefficient. Units were labeled as “unclassed” if either (1) both pattern and component correlations significantly exceed 0, but did not differ significantly from one another, or (2) neither correlation coefficient differed significantly from 0.

To compare the weights MotionNet's V1 and MT units to the synaptic weights connecting V1 and MT neurons proposed by Rust et al. (2006, extracted and replotted from their Fig. 3), we first averaged all weights across speed, so that for each V1 unit there were eight weights corresponding to the eight cardinal/oblique directions. For each V1 unit, we then aligned the averaged weights to the preferred V1 unit direction (see Fig. 3*g*). That is, we rotated the weights of each V1 unit in direction space such that all their preferred directions were aligned to 0°. To further explore the weights, we repeated this process and aligned the weights to either the direction corresponding to the maximum or minimum weight, regardless of V1 unit direction preference (see Fig. 3*h*). Specifically, rather than rotating the weights of each V1 unit in direction space such that all their preferred directions are aligned to 0°, we aligned them such that their maximum (i.e., excitatory) or minimum (i.e., inhibitory) weight was aligned to 0°.

##### Decoding the direction of movement.

**Figure 4.**
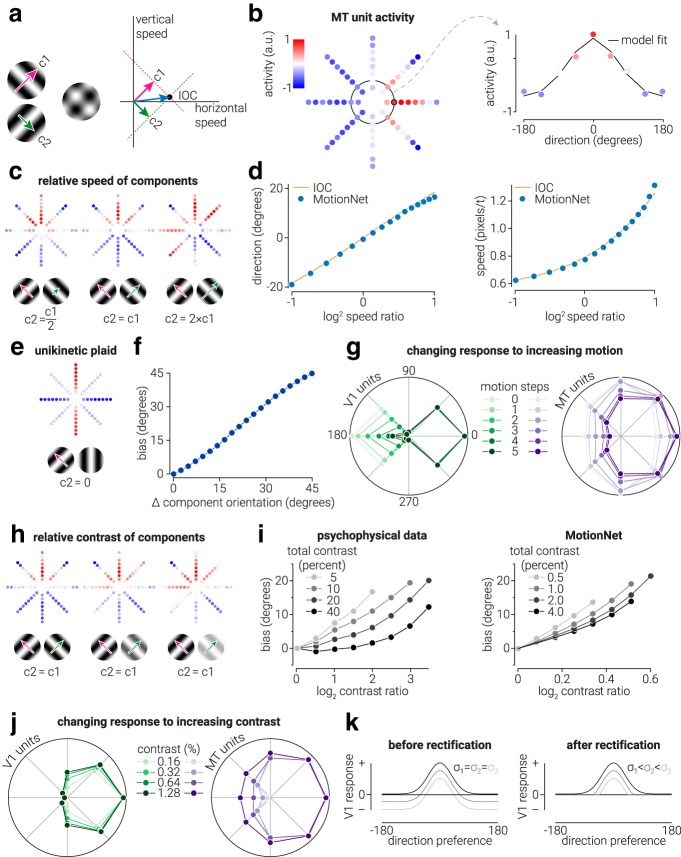
Judging motion direction. ***a***, Intersection of constraints: the true motion of the component gratings could fall anywhere along the dotted constraint lines; their intersection determines perceived velocity. ***b***, MotionNet MT units' response to a rightward-moving plaid. We fit a descriptive model (von Mises function) to the MT units' activity to decode motion as a continuous variable. ***c***, MT unit activity in response to plaids with different speed ratios. ***d***, MotionNet's responses follow the IOC solution over different component grating speed ratios. ***e***, Responses to a unikinetic plaid (one component is static). ***f***, Bias in direction estimates for unikinetic plaids (i.e., bias away from the direction of the moving grating) as a function of the orientation of the static grating. ***g***, Response dynamics in V1 and MT units as a “static” component starts to move (frames 0–5). ***h***, Varying the relative contrast of the component gratings. ***i***, Data from Stone et al. (1990) showing bias in human direction judgments as a function of component contrast ratio at different total contrast levels, and direction decoded from MotionNet. ***j***, The response of V1 and MT units, normalized to the maximum activity, to a moving grating with increasing contrast. ***k***, A diagram illustration showing how V1 unit responses are sharpened by rectification when contrast is reduced.

To compare the velocity estimates from MotionNet to those predicted by the intersection-of-constraints (IOC) rule (see Fig. 4*a*), we tested MotionNet with plaids with a range of component speed ratios (base speed, 0.8 pixels/frame; ratios, 0.5:1 to 2:1 in 16 linear steps) and decoded the direction and speed (see Fig. 4*c*,*d*). The IOC rule velocity was computed as follows:

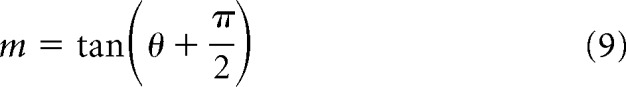
 where θ denotes the angular directions of the two components,

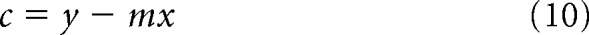
 where *y* and *x* denote the vertical and horizontal velocity of the components, respectively,

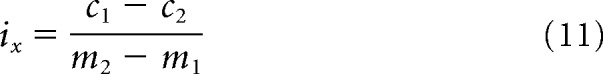
 and

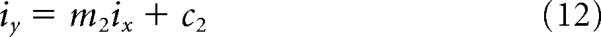
 where *i_x_* and *i_y_* are the horizontal and vertical velocity of the IOC solution, respectively.

When human observers view unikinetic plaids (i.e., plaids comprising one stationary and one moving component), they report perceiving the direction as parallel to the orientation of the static component. To compare human perception with MotionNet's response, we decoded the estimated direction of the network in response to plaid stimuli in which one of the components moved at 3.8 pixels/frame and the other was static, over a range of plaid separation angles (0°-45° in 20 linear steps) (see Fig. 4*e*,*f*). To understand why MotionNet made these estimates, we tested it with sinewave gratings with increasing motion steps, from 0 to 6 (speed, 0.8 pixels/frame) (see Fig. 4*g*). That is, for 0 motion steps, the grating was static, for one motion step the grating moved between the first and second frames then remained static, and so on.

To compare MotionNet's estimates of plaids comprising two components of different contrast with those made by human observers (Stone et al., 1990, their Fig. 11a), we used plaids (speed, 3.8 pixels/frame) in which the relative contrast of the components varied from 0 to 0.6 log contrast ratio in 8 steps. This was repeated at four different levels of total contrast (0.005, 0.01, 0.02, and 0.04), where total contrast is the summed contrast of the two component gratings (see Fig. 4*h*,*i*). To understand why MotionNet was not invariant to the total contrast of the plaids, we tested the network with moving sinewave gratings (speed, 3.8 pixels/frame) at four different contrast levels (0.0016, 0.0032, 0.0064, and 0.0128) and measured the (aligned and averaged) responses of V1 and MT units (see Fig. 4*j*).

##### Interactions between direction and speed tuning.

**Figure 5.**
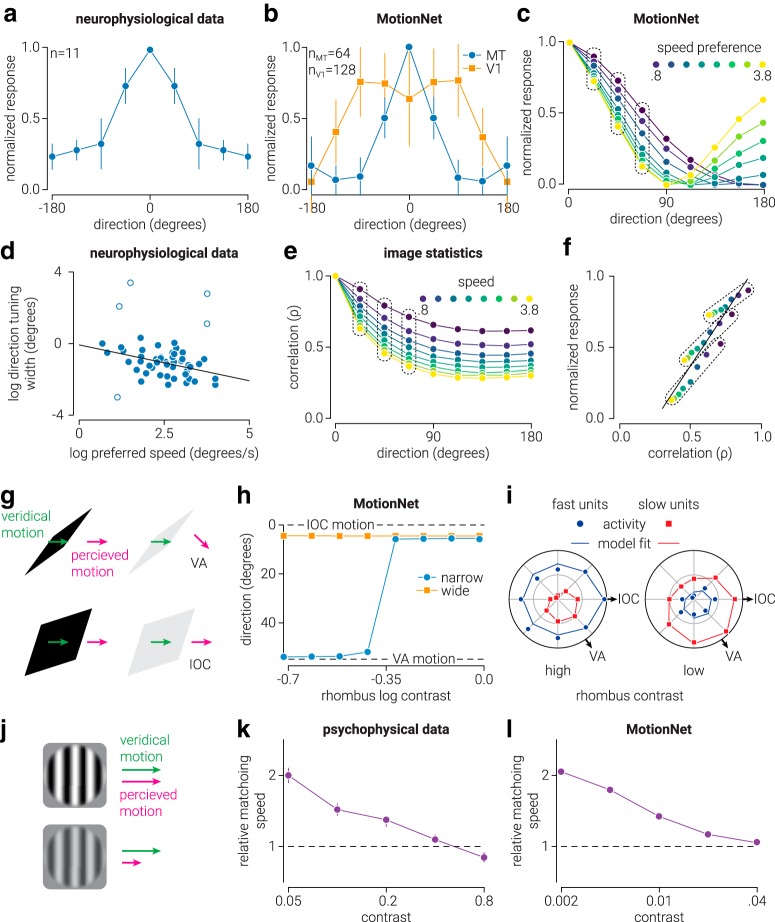
Interactions between misperceptions of direction and speed. ***a***, The average normalized response of macaque MT neurons to motion in the neuron's preferred direction when dots moving in nonpreferred directions are introduced. Data from Snowden et al. (1991). ***b***, The same as in ***a***, but for MotionNet MT and V1 units. Error bars in ***a*** and ***b*** indicate SEM and SD, respectively. ***c***, Motion direction tuning curves of MotionNet MT units with different preferred speeds. ***d***, Reanalysis of data from Wang and Movshon (2016) showing the relationship between speed preference and direction tuning width of macaque MT neurons. Empty circles represent outlier data. ***e***, Similarity analysis showing Pearson's correlation coefficient (ρ) between MotionNet training images moving at direction 0 with the same image moving in other directions, as a function of speed. ***f***, Relationship between the image similarity and MT unit normalized responses indicated by the dotted lines in ***c*** and ***e***. Solid line indicates the linear regression. ***g***, Moving rhombus illusion: a narrow rhombus is perceived in the IOC direction at high contrast but the VA direction at low contrast. ***h***, MotionNet's direction estimates as a function of the rhombus' contrast. Dashed lines indicate IOC and VA solutions. ***i***, MT units, tuned to fast and slow speeds, responding to a moving rhombus presented at high or low contrast. ***j***, Speed bias: the perceived speed of a moving grating is reduced at low contrast. ***k***, Data from Stocker and Simoncelli (2006) showing the relative matching speed of two moving gratings as a function of contrast. Values on the *y* axis indicate how many times faster observers matched the speed of the low-contrast grating to that of the high-contrast grating. Error bars indicate SD of 30 bootstrapped sets of trial data. ***l***, Same as in ***k***, but for speed decoded from MotionNet.

To compare the motion opponency mechanisms (i.e., suppressive activity between V1 and MT) of MotionNet with those found in biological systems (Snowden et al., 1991, data extracted and replotted from their Fig. 11), we tested the network with dot motion stimuli where most (66%) of the dots moved in the preferred direction/speed of a particular V1/MT unit, while the remaining dots moved at the same speed in either the same direction or one of the other seven cardinal/oblique directions. Dot motion stimuli consisted of 12 randomly positioned white dots (pixel value, 1; radius, 5 pixels) on a black background (pixel value, −1), which were allowed to overlap (with occlusion) and wrapped around the image when their position exceeded the edge. We measured the activity of each V1 and MT unit in response to these stimuli before aligning to a common preferred direction and then averaging across all the V1 or MT units (see Fig. 5*b*). The location of maximum suppression was calculated by first taking the average of the responses from the preferred direction to ±180° from preferred direction to create a measure of response as a function of distance from preferred direction; for example, responses at ±45° from the unit's preferred direction were averaged to produce a measure of motion opponency at distance 45°. From these data, we then calculated the distance from the preferred direction of minimum activation.

We established the direction tuning curves shown in Figure 5*c* using the approach described in “Anisotropy in direction of motion responses” above. That is, for each speed, the responses of all MT units tuned to that speed were aligned to the preferred direction and then averaged. We reanalyzed previously collected neurophysiological data (Wang and Movshon, 2016) by correlating the logarithm of direction tuning width and preferred speed using the Pearson correlation, implemented with a correlation analysis MATLAB toolbox (Pernet et al., 2013) (https://sourceforge.net/projects/robustcorrtool/). We tested the normality assumption for the data with the Henze-Zirkler test. The boxplot rule, which relies on the interquartile range (Frigge et al., 1989), was used to reject bivariate outliers (see Fig. 5*d*).

To compare the structure of MotionNet MT units with that of natural image statistics, we computed the Pearson correlation between each natural image sequence from the training set and the same image moving in different directions (0–180°, in 8 steps). This was repeated for each of the speeds (0.8–3.8 pixels/frame, in 8 steps) (see Fig. 5*e*). To assess the relationship between the image statistics and the direction/speed tuning of MT units (see Fig. 5*f*), we computed the Pearson correlation between normalized MT unit responses to stimuli moving in near-to-preferred directions (22.5°, 45°, 67.5°) for all speeds (i.e., responses that defined the width of the MT unit direction tuning curve) with the correlation values between natural images moving in the corresponding directions/speeds.

##### Demonstrations of MotionNet misestimates at low contrast.

To assess MotionNet's estimates of stimuli whose direction is misperceived by humans, we decoded the direction estimated by the network in response to narrow (height-width ratio, 0.1) and wide (height-width ratio, 0.8) moving rhombi for 8 logarithmically spaced contrast levels between 1 and 0.2 (see Fig. 5*g*,*h*). Rhombi were 16 pixels high, oriented 40° clockwise from vertical, moved at 3.8 pixels/frame, and presented on a black background (pixel value, −1). To uncover the basis of MotionNet's misestimates, we visualized the network activity of MT units tuned to the (fast) speed of the rhombus (3.8 pixels/frame) or a slow speed (0.8 pixels/frame), for a thin rhombus presented at either high (1) or low (0.05) contrast (see Fig. 5*i*). To compare MotionNet's speed estimates for stimuli that are misperceived by human observers (Stocker and Simoncelli, 2006, data extracted and replotted from their Fig. 5, top left), we decoded the speed estimated by the network for moving sinewave gratings (speed, 2.5 pixels/frame) at five logarithmically (base 2) spaced contrast levels between 0.002 and 0.04. We compared these with the speed estimated for a sinewave grating moving at full contrast stimulus (see Fig. 5*k*).

##### Relationship between image contrast and speed.

**Figure 6.**
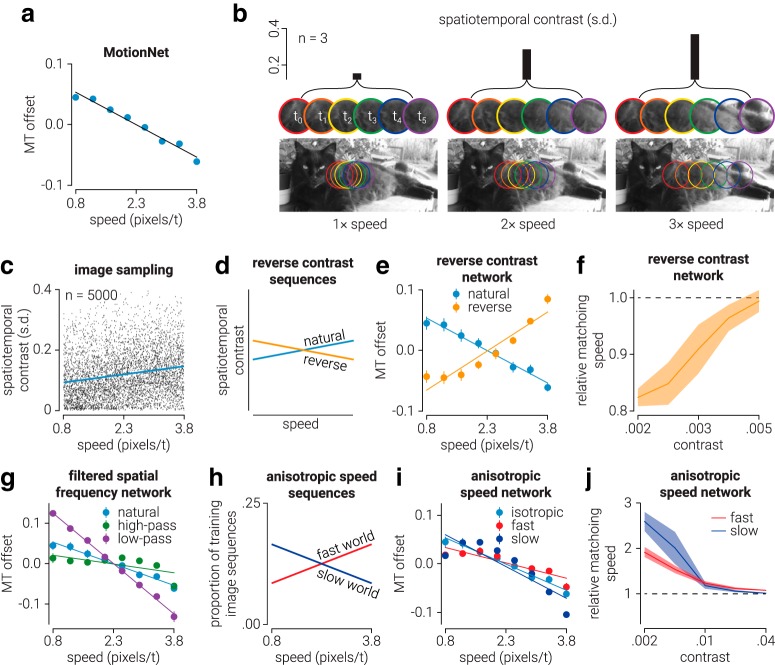
The relationship between image speed and contrast. ***a***, MotionNet MT unit offset parameters as a function of speed tuning, averaged across directions. ***b***, Motion sequence spatiotemporal contrast (indexed by the SD of luminance values across pixels and frames) as a function of speed, for images sampled from a natural image using a moving aperture. ***c***, Spatiotemporal contrast as a function of speed for samples from the training set used for MotionNet. ***d***, Diagram of modifying spatiotemporal contrast to reverse the relationship between motion speed and contrast for a “reverse contrast” network. ***e***, Same as in ***a***, showing data for the reverse contrast network. ***f***, Relative matching speed for the modified network: low-contrast images are estimated as faster. ***g***, Same as in ***a***, but for networks trained on natural images that were blurred (to increase autocorrelation) or sharpened (to reduce autocorrelation). ***h***, The proportions of slow- and fast-moving image sequences used to train “slow world” and “fast world” networks. ***i***, Same as in ***a***, showing data for the “slow world” and “fast world” networks. ***j***, Same as in ***f***, but for the “slow world” and “fast world” networks. ***a***, ***e***, ***f***, ***g***, ***i***, ***j***, Data and error bars/shaded regions represent the mean and SD of 10 network optimizations.

To identify the presence of the low-speed bias in the MT layer of MotionNet, we calculated the Pearson's correlation coefficient of MT unit offset parameters (averaged across preferred direction) as a function of preferred speed (see Fig. 6*a*). For the V1 layer units, we identified the presence of the bias by computing an independent *t* test between the offset parameter of V1 units with maximum weights connected to either “slow-preferring” (i.e., the lowest four speeds) or “fast-preferring” (i.e., the highest four speeds) MT units. For this analysis, we ordered V1 units by their absolute weight and only considered the top 50% most influential V1 units (units with near 0 weights had very little influence on the network's performance).

To illustrate the relationship between image contrast and speed, we computed the spatiotemporal contrast of sections of a natural image captured within an aperture moving at three different speeds (see Fig. 6*b*). Spatiotemporal contrast was defined as the SD of all pixel values within the motion sequence (i.e., the SD across *x*, *y*, *t* of the image sequence); this is similar to root mean squared contrast across time, and the same results are found using either measure. To systematically investigate the relationship between spatiotemporal contrast and movement speed, we sampled 5000 natural image sequences (taken from those used to train MotionNet). Images (16 × 16 × 6 pixels) moved in the same (rightward) direction at randomly selected speeds between 1 and 16 pixels/frame. We then computed the Pearson correlation between speed and the spatiotemporal contrast of each sequence (see Fig. 6*c*).

To test the prediction that the slow-speed bias observed in MotionNet was a product of the relationship between image speed and spatiotemporal contrast present in natural image sequences, we modified the natural image sequences used to train MotionNet to artificially reverse this relationship (see Fig. 6*d–f*). We first calculated the strength of the relationship in the natural images used to train MotionNet. We then applied the inverse of this relationship to the images, increasing the luminance contrast of slow image sequences and reducing the contrast of fast sequences. Image contrast was increased/decreased by multiplying/dividing the value of all pixels within an image sequence around the average pixel value. This modified training set was then used to train the reverse contrast network. As a further test, we manipulated the degree of spatial autocorrelation between pixels by blurring (convolution with a Gaussian with SD = 20 pixels) or sharpening (convolving with inverted form of the blurring Gaussian) the natural image sequences, and training new networks on these modified training images (see Fig. 6*g*).

To test the idea that the perceptual speed bias observed in human observers when viewing low-contrast moving images is the product of a prior, which reflects the anisotropic distribution of speeds in the environment (i.e., the “slow world” prior) (Weiss et al., 2002; Stocker and Simoncelli, 2006), we trained two new networks on image sequences in which the distribution of speeds was uneven. For the “slow world” network, the proportion of image sequences as a function of speed decreased linearly such that there were twice as many slow speeds as fast speeds included in the training set. For the “fast world” network, the opposite was true (see Fig. 6*h*,*i*). For the reverse contrast (see Fig. 6*f*) and “slow/fast world” networks (see Fig. 6*j*), we used the same method for the simulation of the standard MotionNet network (see Fig. 5*k*) with the exception that the contrast levels used for the reverse contrast network were between 0.0015 and 0.005. The contrast range that best demonstrated the bias was different in the reduced contrast network as a result of having altered the contrast of the training images.

##### Psychophysical experiment.

*A priori* sample size was established using the effect size from Stocker and Simoncelli (2006) to achieve >90% power. Four female and four male human participants (age, 27.6 ± 4.7 years) with normal or corrected-to-normal vision (tested using a Snellen chart) participated. Experimental procedures were approved by the University of Cambridge Ethics Committee. Observers provided written informed consent. Seven subjects were naive to the purpose of the study.

Stimuli were generated in MATLAB using Psychtoolbox extensions (Brainard, 1997; Pelli, 1997) and displayed on a calibrated ViewPixx monitor (VPixx Technologies) running at a framerate of 120 Hz and an average background luminance of 84 cd/m^2^. Participants viewed the visual display binocularly from a distance of 58 cm, using a chin rest to stabilize their head position.

The experimental procedure was similar to that used by Stocker and Simoncelli (2006). Subjects were presented with two circular patches containing horizontally drifting sinewave gratings (spatial frequency, 1°/cycle; random start phase). Patches were 2.5° in diameter, centered 6° either side of the fixation dot. The mean luminance of both gratings was held constant at 84 cd/m^2^. Subjects were instructed to fixate a central fixation dot while each stimulus was presented for 700 ms. Following stimulus presentation, subjects selected the stimulus that appeared to be moving faster, on average, during the presentation. Response duration was unrestricted.

Each pair of stimuli consisted of a reference and a test grating that were assigned to the left and right presentation locations. On each trial, both gratings moved in the same direction (left or right). The reference grating had a contrast of 0.5. Three different types of test gratings (baseline, low-contrast, and variable speed) were interleaved throughout each block. The baseline test grating was identical to the reference test grating, the low-contrast grating had a contrast of 0.1, and on each frame the speed of the variable speed grating was altered by a pseudo-randomly selected value from a Gaussian distribution (mean, 0; SD, 0.2°/cycle). Importantly, we ensured that the average speed, across the presentation, of the variable speed grating was not altered by the manipulation. The speed of the reference grating was 1°/cycle, and the baseline and variable speed test gratings moved at 1 of 7 evenly spaced speeds between 0.25 and 1.75°/cycle, and the low-contrast test grating moved at 1 of 7 evenly spaced speeds between 0.65 and 2.15°/cycle (method of constant stimuli procedure). The speed range was established during pilot testing. Each participant completed nine blocks, with each block comprising 168 trials. Test grating location, direction, condition, and speed were all pseudo-randomly selected on each trial, in a counterbalanced manner across each block. Psychometric functions were fit with Psignifit (Fründ et al., 2011). Speed bias was derived from the threshold of the psychometric function and estimate uncertainty from the slope.

##### Simulation of experimental predictions.

**Figure 7.**
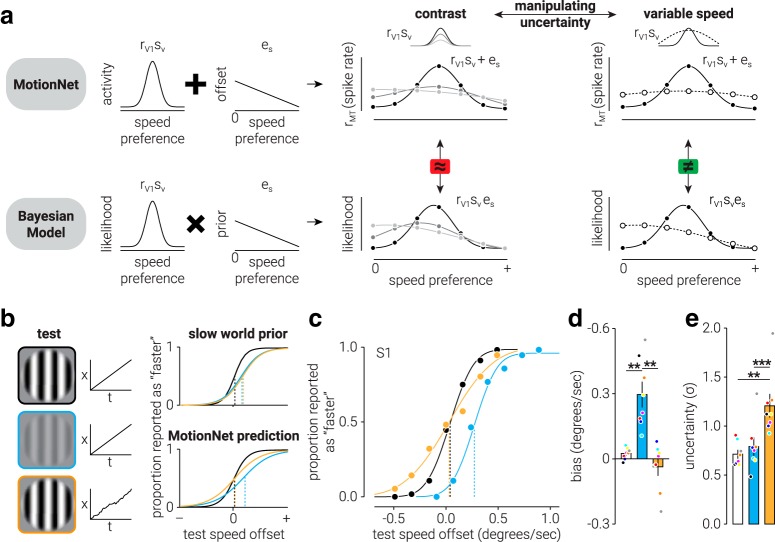
Motion models and psychophysical tests of MotionNet predictions. ***a***, Illustration comparing speed estimation by MotionNet versus a “slow world” prior model. Left, MotionNet represents speed as a distribution of activity (r_V1_), which is summed with a constant offset (e_s_) to produce the final estimate (r_MT_). The Bayesian model takes the product of the likelihood and prior distributions. Right, Uncertainty can be manipulated by reducing image contrast or introducing variability in speed. For contrast, uncertainty is increased while signal amplitude is reduced, so MotionNet and the Bayesian model make equivalent predictions. For speed variability, uncertainty is increased while signal amplitude remains unchanged, and MotionNet and the Bayesian model make divergent predictions. ***b***, Left, Illustrations of test stimuli, and their speed profiles, used in the psychophysical experiment. Right, Simulations showing the predictions made by the two models. ***c***, Results of a representative human observer. ***d***, ***e***, Summary results of all observers for (***d***) speed bias and (***e***) estimate uncertainty. Separate repeated-measures ANOVA tests revealed main effects of condition for both speed bias (*F*_(2,14)_ = 14.37, *p* = 0.005) and uncertainty (χ^2^_(2,14)_ = 12.25, *p* = 0.002). Colors represent data from conditions shown in ***b*** (left). ***d***, ***e***, Dots indicate individual datum. Colors represent corresponding observers. Error bars indicate SEM. **p < 0.01, ***p < 0.001.

To generate MotionNet predictions of speed in response to low-contrast or variable speed stimuli (see Fig. 7*a*), we calculated the following:

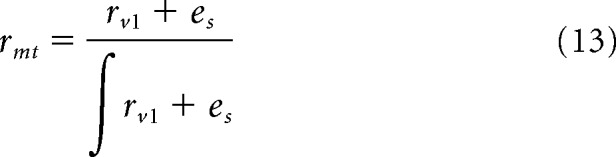
 where *r_mt_* is the MT unit population response, *r_vl_* is the response from V1, and *e_s_* denotes the low-speed bias. The low-speed bias was defined as a linear gradient with maximum at speed 0 (maximum value = 0.5, slope = −0.5); however, the same pattern of results is found if a Gaussian prior centered on 0 is used. The response of each MT unit is divided by the sum of activity across the population to represent divisive normalization (Heeger, 1992). For reduced contrast simulations, three different V1 responses were simulated, intended to represent different levels of contrast. In particular, *r_vl_* was defined as a Gaussian distribution where the offset (*p*) was 0, the mean (*x*_0_) was 1, and the widths (σ) and amplitudes (*A*) were [0.3, 0.6, 1.2] and [1, 0.2, 0.15], respectively.

For variable speed simulations, we used the same parameters as used in the first and last *r_vl_* of the reduced contrast simulation (i.e., high and low contrast), except that the same *A* was used for both (i.e., 1). This was intended to represent the manipulation of signal uncertainty without the changing amplitude. The “slow world” prior predictions were computed using the same parameters, except that *r_vl_* and *e_s_* were combined through multiplication, rather than addition as follows:

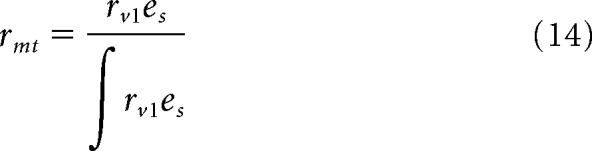
 To generate predictions of the experimental results, we defined three Gaussian distributions (one for each of the conditions in the psychophysical experiment) (see Fig. 7*b*). Distributions were created using Equation 6, where the offset (*p*) was 0 and the mean (*x*_0_) was 0.5. The baseline distribution had an amplitude (*A*) of 1 and a width (σ) of 0.2. The reduced contrast distribution had a lower amplitude (0.05) and larger width (0.5) than the baseline, to reflect the reduced activation and lower certainty of a low-contrast moving stimulus. The variable speed distribution had the same amplitude as the baseline distribution and the same width as the reduced contrast distribution, to reflect selectively reduced certainty in this condition. We modeled the low-speed bias as a linear gradient with maximum at speed 0. For the “slow world” prior simulations, we combined the conditional distributions with the bias distribution through multiplication; and for the “reduced contrast prior,” we combined these through addition. To generate psychometric functions, we simulated the psychophysical experiment using the “slow world” and “speed-contrast” distributions. For each condition, we simulated 20 speed offsets between −1 and 1 on 500 simulated trials. On each trial, we pseudo-randomly sampled one speed value from the baseline distribution and one value from one of the three condition distributions (baseline/reduced contrast/variable speed). We then summed the offset value with the value sampled from the conditional distribution and determined whether it was larger than the baseline sampled value. From this, we were able to plot the proportion of trials in which the conditional value was larger than the baseline value as a function of speed offset, and used Psignifit to fit a psychophysical function to obtain threshold values.

##### Data reanalysis.

Data in Figures 2*a*, *i*, 3*d*, *f*, 4*i*, and 5*a*, *d* were extracted from previous studies (Movshon et al., 1983; Stone et al., 1990; Snowden et al., 1991; Matthews and Qian, 1999; Rust et al., 2006; Stocker and Simoncelli, 2006; Salinas et al., 2017) using WebPlotDigitalizer (automeris.io/WebPlotDigitizer). Data in Figure 5*d* are a reanalysis of archived data (https://archive.nyu.edu/handle/2451/34281) from Wang and Movshon (2016).

##### Significance testing.

For analysis of the psychophysical data, we used the repeated-measures ANOVA and *t* test; all tests were two-sided. We first used repeated-measures ANOVAs to test for main effects; we then followed up with *t* tests as appropriate to determine the precise relationship between conditions. The normality and sphericity assumption was tested with the Shapiro–Wilk test of normality and the Mauchly's test of sphericity. For speed bias, but not estimator uncertainty, the assumption of sphericity was violated; thus, we used the Greenhouse–Giesser-corrected *F* value. For estimator uncertainty, the distribution in the variable speed condition was found to violate the assumption of normality. Thus, a nonparametric repeated-measures ANOVA (Friedman test) was used to test for main effects of uncertainty. To determine the significance of data generated by MotionNet, we used independent-samples *t* test and the Pearson's correlation.

##### Data availability.

We performed analyses in Python using standard packages for numeric and scientific computing. All the code and data used for model optimization, implementations of the optimization procedure, and behavioral data are freely and openly available at www.repository.cam.ac.uk/handle/1810/300898.

## Results

### Network architecture and training

We created a neural network (MotionNet) tasked with decoding motion (Fig. 1*b*). The input was a sequence of image frames (*x-y*) depicting a scene moving through time (*t*). This was convolved with 3D kernels (*x-y-t*), analogous to spatiotemporal receptive fields in area V1. This activity was read out by decision units selective to direction and speed, analogous to MT/V5 neurons.

We trained MotionNet to classify the velocity of natural images moving with 8 different speeds (0.8–3.8 pixels/frame) in 1 of 8 directions (4 cardinal; 4 oblique). Following training, it could classify novel sequences with high accuracy (∼80% vs chance = 1.56%). V1 units were initialized with Gaussian noise, but after training they resembled (Fig. 1*c*) receptive fields in primary visual cortex (Movshon et al., 1978; Rust et al., 2005). Although MotionNet was trained on translating natural images, its performance generalized to complex movies comprising optic flow and object motion (∼50% accuracy vs 1.56% chance) as well as sinusoidal gratings (accuracy = 100%).

### What is the basis for estimating motion direction?

We start by examining the direction preferences of MotionNet's V1 units. Biologically, more V1 neurons respond to cardinal than oblique motion directions (Salinas et al., 2017) (Fig. 2*a*). Intuitively, this might reflect past experience: movement over the ground, and the effects of gravity, would cause biological systems to experience more cardinal motion. However, we observed nonuniform preferences in MotionNet (Fig. 2*b*) despite being trained on a uniform distribution of directions.

What accounts for this difference between the direction distribution of the training set and MotionNet? We hypothesized that the dominance of cardinal orientations in natural images (Girshick et al., 2011) might be responsible. We therefore trained a new network using images rotated by 45°. In line with our reasoning, this network had a preference for oblique directions (Fig. 2*c*). As a control, we trained another network on random dot patterns (which convey no systematic orientation). In this case, V1 unit preferences were uniformly distributed, mirroring the direction statistics of the training set (Fig. 2*d*). However, biased distributions were restored if dots were distorted in the cardinal (Fig. 2*e*) or oblique (Fig. 2*f*) directions. We infer that the overrepresentation of cardinal motion directions in V1 neurons is likely to be explained by natural image orientations per se, rather than motion statistics.

What are the consequences of V1 direction anisotropy? We examined MT units' sensitivity to cardinal versus oblique directions, finding sharper tuning for cardinal directions (Fig. 2*g*). This produces greater sensitivity for cardinal motion (Fig. 2*h*), mirroring human perceptual judgments (Matthews and Qian, 1999) (Fig. 2*i*).

### How are motion signals integrated to estimate direction?

Judging an object's movement depends on integrating signals from across the stimulus as local motions are often ambiguous (“the aperture problem”; Fig. 1*a*, blue bars). For instance, the barber pole's illusory motion results from integrating ambiguous signals from the center with disambiguated signals from the edges. Consistent with human perception (Fisher and Zanker, 2001), MotionNet estimated horizontally translating stripes as moving upward, contingent on the width of the “pole” (Fig. 3*a*,*b*). Inspecting MotionNet's V1 units revealed that activity at the center signaled motion orthogonal to the stripes, whereas activity at the edges signaled motion either parallel to the stripes or in the opposite direction. Pooling of this information by MT units produces an upward motion estimate.

Experimental tests of motion integration often use plaid patterns composed of two sinewave components (Fig. 3*c*). The individual components can move in different directions from the overall plaid (Adelson and Movshon, 1982) and V1 neurons signal motion of the components (Movshon et al., 1983; Gizzi et al., 1990). For example, the V1 neuron shown in Figure 3*d* responds most strongly to a leftward moving grating; but when shown a plaid, it responds most strongly to motion above or below leftward such that one of the component gratings moves leftward. The same behavior is observed for MotionNet V1 units (Fig. 3*e*). By contrast, MT neurons show pattern-motion selectivity (Fig. 3*d*, bottom), responding to the plaid's features, rather than the individual components. MotionNet's MT units showed the same behavior (Fig. 3*e*).

This shows that training MotionNet to classify movement using brief natural image sequences reproduces key properties of biological V1 and MT (Fig. 3*d*,*e*). MotionNet's units were classified as component- or pattern-selective more consistently than neurons (Movshon et al., 1983). Higher noise within the biological system may make true tuning preferences harder to reveal.

Having identified strong parallels between the artificial and biological system, we next studied how V1 component-selective activity is transformed into MT pattern selectivity. Previously, a readout scheme was proposed with maximum excitatory connections between V1 and MT units of the same direction preference (Rust et al., 2006) (Fig. 3*f*). However, when we organized MotionNet's connections according to V1 preferences, the average weights were primarily inhibitory with bimodal peaks ±90° from the preferred direction (Fig. 3*g*). Why might this be so? To find out, we ordered inputs according to the maximum or minimum weight, without consideration of V1 unit preferences. That is, rather than aligning the preferred directions of each V1 unit in direction space to 0°, we aligned them such that their maximum (i.e., excitatory) or minimum (i.e., inhibitory) weight is aligned to 0°. Whereas the preferred direction of a V1 unit is defined by the stimulus motion direction to which it is maximally excited, the maximum and minimum weights correspond to the preferred directions of the MT units to which it most excites or suppresses, respectively. Ordering by the maximum weight revealed a function resembling a second derivative Gaussian (Fig. 3*h*, red line). This pattern of connections makes intuitive sense: it promotes activity of MT unit's preferred direction while inhibiting nearby directions. However, activity organized by the minimum weight (Fig. 3*h*, blue line) revealed a pattern similar to aligning with the V1 preference (Fig. 3*g*). This shows that the network acts primarily to inhibit nonpreferred directions, rather than promoting the preferred direction. Specifically, a V1 unit's preferred direction is more predictive of maximum inhibition of the MT unit (i.e., in nonpreferred directions) than maximum excitation in the preferred direction. This explains the local suppressive trough when weights are aligned to V1 unit preference (Fig. 3*h*): the opposite direction drives inhibition resulting in stronger suppression. More generally, this suggests that the properties of V1 direction selectivity are better understood in relation to their readout by MT units, rather than the way in which their direction properties have been functionally characterized. In particular, the specificities ascribed to V1 and MT units may look equivalent within the context of a particular parameter space (e.g., movement direction), but the computational purposes of V1 versus MT representations will be different. While it is intuitively appealing to map a preferred response to a given stimulus across layers of a network, this can provide a misleading impression of their computational purposes. For instance, we previously showed (in a different context) that V1 responses are better understood as providing evidence for and against particular interpretations of a scene, rather than acting as detectors for a particular binocular disparity (Goncalves and Welchman, 2017; Rideaux and Welchman, 2018).

### Why is motion direction misperceived?

Having found strong parallels with neurophysiology, we next examined similarities to perception by manipulating plaids in ways that bias human judgments away from the physical motion direction. Although the perception of a plaid's direction typically follows the IOC rule (Adelson and Movshon, 1982) (Fig. 4*a*), this does not always occur. We tested MotionNet with a variety of plaids and examined its estimates (Fig. 4*b*).

First, we studied direction estimates by varying the component gratings' speeds (Fig. 4*c*). We found that MotionNet's estimates, like perception, follow the IOC (Fig. 4*d*). We then tested “unikinetic” plaids, in which one component is static (Gorea and Lorenceau, 1991). This is interesting because perceived direction varies, even though the physical direction remains unchanged. Consistent with human perception, MotionNet estimated the direction of a unikinetic plaid as parallel to the static component's orientation (Fig. 4*e*,*f*). To understand why, we titrated movement of the “static” component. We found that a static grating evokes the most activity from V1 units with preferred directions orthogonal (e.g., leftward and rightward) to its orientation (e.g., vertical); this produces mutual inhibition of MT units with preferred leftward and rightward directions so that there is no overall excitatory contribution to the direction estimate (Fig. 4*g*). However, as the grating begins to move, activity of V1 units preferring the opposite direction (e.g., leftward) collapses, releasing MT units tuned to rightward motion from inhibition, and thereby shifting the peak of activity toward the right. Thus, static gratings inhibit MT units tuned to directions orthogonal to their orientation, biasing the direction of the moving component so that it is seen moving parallel to the static component. In biological systems, this inhibition may affect neuronal gain, rather than directly suppressing the firing rate of MT neurons (Wallisch and Movshon, 2019) (i.e., inhibition that affects gain without evoking a suppression).

Finally, we tested the relative contrast of the components (Fig. 4*h*) as humans show a perceptual bias toward the component with higher contrast (Stone et al., 1990). Mirroring human judgments, MotionNet's estimates were biased away from the IOC solution and varied as a function of total contrast (Fig. 4*i*). This was unexpected. In particular, we anticipated that MotionNet's direction estimates might be influenced by the contrast ratio, as this could lead to more activity in one component direction than the other. However, we expected the artificial system, unlike biological systems, to be largely invariant to the absolute contrast of the stimulus when the relative contrast of the components was held constant (the relationship between luminance contrast and neuronal response is nonlinear; Albrecht and Hamilton, 1982), whereas MotionNet applies no compressive or expansive nonlinear computations to the image. To understand why this occurs, we tested gratings with increasing contrast. At low contrast, gratings produced narrower peaks of activity across V1 and MT units than at high contrast (Fig. 4*j*). The difference in peak width is due to signal rectification. That is, when stimulus contrast is low, the activity of V1 units tuned to the nonpeak direction is rectified (Fig. 4*k*, left), which sharpens the response that is transmitted to the MT units (Fig. 4*k*, right). Thus, when total contrast is reduced, the activity produced by the two components comprising the plaid becomes narrower, which results in greater dominance of the stronger (high-contrast) component when combined.

### Interactions between direction and speed processing

We have seen that inhibition plays an important role in shaping responses to plaids. To test inhibitory responses *in vivo*, experimenters have presented moving dot patterns and then overlaid dots moving in a different direction. V1 neurons are not substantially affected by this manipulation; however, MT neurons show motion opponency and are suppressed by dots moving in a nonpreferred direction (Snowden et al., 1991; Qian and Andersen, 1994; Rust et al., 2006) (Fig. 5*a*). We found comparable responses within MotionNet: V1 units are only weakly affected, whereas MT units show motion opponency (Fig. 5*b*). We then tested the relationship between speed preferences and the direction that evoked maximal suppression. There was a weak relationship for V1 (*n* = 128, ρ = −0.21, *p* = 0.02), but a strong relationship for MT (*n* = 64, ρ = −0.87, *p* = 1.9e^−20^): MT units tuned to fast speed are maximally suppressed by closer-to-preferred directions than slow speed units. In particular, MotionNet learnt sharper direction tuning for fast speed MT units (Fig. 5*c*).

This set up a clear prediction to test against biological data. We reanalyzed a neurophysiological dataset (Wang and Movshon, 2016), finding that direction tuning and speed preference are related for macaque MT neurons (*n* = 48, ρ = −0.45, *p* = 0.001; Fig. 5*d*). Specifically, MT neurons tuned to fast speeds have sharper direction tuning than those tuned to slow speeds. Why does direction tuning sharpen with speed? We analyzed the image statistics of MotionNet's training set and found that, as speed increases, the similarity between images moving in different directions is reduced (Fig. 5*e*). Thus, a slow-moving sequence will produce more activity in adjacent-to-preferred directions of direction-selective units with the result that tuning curves are broader. Formally, image similarity is highly correlated with MT unit direction tuning (*n* = 24, ρ = 0.93, *p* = 4.6e^−11^; Fig. 5*f*); this may explain previous neurophysiological evidence suggesting that motion opponency in MT is related to speed tuning (Krekelberg and van Wezel, 2013). To rule out the possibility that fast-moving images evoked activity in the opposite direction due to spatiotemporal aliasing, we trained and/or tested new networks on lowpass filtered images, finding the same pattern of results.

Human observers' ability to discriminate between directions improves with increasing speed, over a wide range of speeds (0.5–64 deg/s) (De Bruyn and Orban, 1988). This has been attributed to the static orientation information that is produced by sensory persistence of fast-moving stimuli (i.e., motion streaks) (Geisler, 1999; Tong et al., 2007). However, there is no persistence that could produce something equivalent to motion streaks within MotionNet, yet the network develops the same relationship between direction and speed tuning. This result does not preclude the role of sensory persistence in supporting human motion perception, but it shows that the spatial autocorrelation in natural images could be sufficient to explain the relationship between direction and speed at the perceptual and neurophysiological level.

Next, we explored why human observers are susceptible to illusions of direction and speed using two classic examples. First, when a rhombus moves to the right (Fig. 5*g*), the IOC solution from its edges indicates rightward motion. However, the vector averaging (VA) solution (the mean of the two motion vectors from the edges) is consistent with diagonal motion down and to the right. If the rhombus is wide or the contrast high, observers' judgments follow the (veridical) IOC solution. However, if the rhombus is narrow and low contrast, observers report the (false) VA direction (Weiss et al., 2002). MotionNet exhibited the same pattern (Fig. 5*h*): a narrow rhombus is estimated in the IOC direction at high contrast, and the VA direction at low contrast. However, a wide rhombus is estimated in the IOC direction even at low contrast. Why does this occur? MT units tuned to fast speeds decode the rhombus' motion in the IOC direction, but units tuned to slower speeds decode its motion in the VA direction. When the rhombus is presented at high contrast, the fast units are most active; but at low contrast, the slow units become more active and the decoded motion shifts from the IOC to the VA direction (Fig. 5*i*). To the best of our knowledge, physiological data on this motion illusion have not yet been published. Weiss et al. (2002) provide a theoretical Bayesian model to explain the illusion, whereas here we provide a biologically explicit explanation. Future work could test this prediction by comparing the activity of MT neurons tuned with slow or fast motion in response to these stimuli.

This demonstrates how perceived direction can be biased at low image contrast. However, misperceptions of speed also occur: low-contrast gratings appear to move slower (Hürlimann et al., 2002; Stocker and Simoncelli, 2006) (Fig. 5*j*,*k*). Consistent with human judgments, we found that MotionNet computed the speed of a moving grating as slower as its contrast was reduced (Fig. 5*l*). We next explain why image contrast is a critical variable when perceiving motion.

### How is the speed of movement estimated?

The illusions in Figure 5 were previously explained by a Bayesian model that assumes humans internalize the motion statistics of the environment. It was suggested that net motion of the environment is close to 0 (a “slow world” prior), and that this informs motion judgments (Weiss et al., 2002; Stocker and Simoncelli, 2006). Under the Bayesian framework, the influence of a prior becomes more apparent when the stimulus is uncertain. Specifically, lowering image contrast reduces the reliability with which motion is estimated, increasing the prior's influence (centered on 0 speed) and thus biasing perception toward slower motion. If bias toward slow motion results from natural motion statistics, MotionNet should not reproduce these phenomena as the training set contained an equal distribution of speeds. However, Figure 5 shows that lowering contrast results in slower speed estimates for humans and MotionNet. Why?

MotionNet consists of weight parameters, which are multiplicative, and offset parameters, which are additive/subtractive. As the contrast of the image is reduced, so too is the influence of the weights, while the influence of the offsets remains constant. Thus, reducing contrast increases the relative influence of the offset parameters. Inspecting MotionNet's offset parameters shows a clear trend favoring low speeds (MT: *n* = 8, ρ = −0.99, *p* = 4.3e^−6^; Fig. 6*a*; V1: *n* = 64, independent *t* test = 6.95, *p* = 2.8e^−9^, *d* = 1.75). As the proportion of motion speeds used to train the network was equal, bias cannot be the network's response to an environment with more slow speeds. Instead, the network learnt a relationship between contrast and the speed of moving images.

Luminance values in natural images tend to be highly correlated between adjacent pixels as physical objects are typically contiguous in form and reflectance (the basis for efficient encoding) (Attneave, 1954; Barlow, 1961; Simoncelli and Olshausen, 2001). This association also has implications for motion (Dong and Atick, 1995). Figure 6*b* illustrates an image with samples extracted from an aperture moving at different speeds: spatiotemporal contrast (indexed by the SD of luminance values across pixels and frames) becomes higher as speed increases. Specifically, the relationship between luminance and proximity dictates that, when the distance between samples is low, they will be more correlated and thus have lower spatiotemporal contrast, than when the distance between samples is high. To demonstrate this relationship, we sampled motion sequences for a range of speeds using natural images. We found a positive relationship between speed and spatiotemporal contrast (*n* = 5000, ρ = 0.19, *p* = 1.6e^−43^; Fig. 6*c*).

To test whether the speed-contrast association accounts for MotionNet's slow speed estimates, we trained a new network on sequences in which the relationship was artificially reversed (Fig. 6*d*); that is, low-speed sequences were altered to have higher spatiotemporal contrast than high-speed sequences. Consistent with our hypothesis, the offset parameters learned by this network were reversed, showing a bias for high speeds (*n* = 8, ρ = 0.95, *p* = 3.2e^−4^; Fig. 6*e*). We repeated the speed matching experiment and found that low-contrast images were now judged as faster (Fig. 6*f*).

If the correlation structure of the viewed images is critical, we reasoned that modifying spatial correlation should alter the association between speed and contrast. Specifically, we created stimuli that were either blurred or sharpened versions of the original images. This has the effect of increasing or reducing the images' spatial autocorrelation. We trained new networks with these stimuli, finding that the slow-speed bias was increased for the network trained on blurred stimuli, but reduced for the network trained on sharpened images (Fig. 6*g*). Thus, the correlation structure of natural images appears critical in determining motion estimation.

As a further test, we instantiated new networks trained under “slow world” or “fast world” conditions. We trained these networks on a higher proportion of either slow or fast speeds of motion, while leaving the spatial autocorrelation and spatiotemporal contrast of the images unaltered (Fig. 6*h*). This directly pitted the notion of motion statistics against our hypothesis regarding spatiotemporal contrast. Consistent with MotionNet, both networks showed a bias toward slow speeds for low-contrast stimuli. While changing the distribution of speeds presented to the network will change what it learns to some extent, these results indicate that contrast, rather than “slow world” motion statistics, is central to the phenomenon (Fig. 6*i*,*j*).

Could these results be explained by motion energy mechanisms? The original motion energy model (Adelson and Bergen, 1985) was sensitive to image contrast, but with biases much larger than those observed psychophysically (Stocker and Simoncelli, 2006). Subsequent revisions of the model (Heeger, 1987) include normalization that renders the model largely invariant to contrast. This allows the energy model to accurately predict the motion of plaids with high component contrast ratios; however, it fails to capture perceptual biases.

### Psychophysically testing predictions derived from MotionNet

Previous work showed that humans underestimate the speed of a low-contrast stimulus (shift in subjective equality between test and reference stimuli), and their responses are more variable (shallower psychometric function) (Hürlimann et al., 2002; Stocker and Simoncelli, 2006). We used this paradigm to pit the predictions of a “slow world” prior model against our hypothesis about internalized knowledge of the speed-contrast relationship (Fig. 7*a*). We developed a paradigm to reduce sensory reliability without manipulating image contrast. We reasoned that, if observers rely on a speed prior, reducing sensory reliability in any way should increase the prior's influence, with the result that movement is perceived as slower. However, if image contrast is the key variable, a reliability manipulation that did not alter contrast should not affect speed judgments. The key differentiating principle between these computations is that MotionNet sums the offset with activity while the Bayesian model takes their product (Fig. 7*a*).

We compared speed matching performance between three conditions: the test stimulus was (1) baseline: the same as; (2) low contrast: of lower contrast; or (3) variable speed: had variable speed compared with the reference stimulus (Fig. 7*b*) (Bentvelzen et al., 2009). If the slow prior is general, we expected the same results for both types of motion uncertainty. However, if the prior is restricted to low contrast, we would not expect bias in the variable speed condition.

Figure 7*c* shows the results of a representative observer. Consistent with previous work, low-contrast stimuli appear slower (*n* = 8, paired *t* test = 4.64, *p* = 0.002, *d* = 1.64; Fig. 7*d*); however, we found no evidence for bias in the variable speed condition (*n* = 8, paired *t* test = 1.50, *p* = 0.176, *d* = 0.53) relative to baseline. Directly contrasting low contrast against variable speed conditions showed larger bias in the low-contrast condition (*n* = 8, paired *t* test = 3.72, *p* = 0.008, *d* = 1.31). Importantly, observers' responses were more variable (i.e., shallower slope of the psychometric function) in the variable speed condition than in the low-contrast condition (*n* = 8, paired *t* test = 7.38, *p* = 1.5e^−4^_,_ *d* = 2.61; Fig. 7*f*), meaning that the motion prior framework would predict a stronger bias for the variable speed condition. These results are consistent with the interpretation that the slow-speed bias is specific to low contrast and support the idea that the human visual system learns the relationship between speed and spatiotemporal contrast.

## Discussion

The ability to perceive movement is a key foundation of vertebrate evolution (Walls, 1942). Human misperceptions are therefore surprising, and it is challenging to understand how these emerge from evolution and experience. Here we use an artificial system to understand the drivers that shape motion estimation: a neural network trained on moving images recapitulates key physiological and perceptual properties of biological brains. Using complete access to the system, we propose why neurophysiological properties emerge and the basis for misperceptions, as well as making concrete predictions for future empirical study. Our results recast understanding of the statistics that are internalized by the brain.

Importantly, the convergence we demonstrate between MotionNet and biology was far from certain *a priori*. While the network's architecture is loosely based on the brain, the similarities are dwarfed by the differences (e.g., no eye movements or recurrent connections; presence of an explicit training signal; to name but a few). Moreover, once MotionNet was trained on natural images, its properties were fixed: subsequent tests using laboratory stimuli (gratings, plaids, random dots) on a range of tasks referenced to both neurophysiology and perception formed no part of the training objective. The parallels we observe between the network and biology, despite manifold differences, therefore point to the common element between them: the image structure that optimizes visual systems. Specifically, we show that image statistics determine encoding properties, interrelations between movement direction and speed, spatiotemporal contrast, and the relationship between speed and contrast.

### Neural nets can advance neuroscience: specific predictions for future experiments

There have been notable artificial intelligence successes in solving tasks performed by the brain, from perception (Krizhevsky et al., 2012) to cognition (Sutskever et al., 2014) and motor control (Ali Eslami et al., 2018). However, the complexity of deep networks makes it hard to interpret their operation and inform neuroscience. The opportunity of using artificial systems is to use complete access to track the chain of operations. Here, we purposefully implemented a “shallow” network (one hidden layer), allowing us to interrogate the relationship between inputs and outputs. We successfully tested novel predictions against neurophysiological and perceptual data. However, we also make concrete predictions for future experiments:

1. Excitatory and inhibitory projections from V1 neurons to MT neurons resemble a second derivative Gaussian as a function of direction offset. Further, a V1 neuron's preferred direction is more predictive of maximal inhibition of MT in the opposite direction, rather than maximal excitation.
2. A static grating evokes inhibition in MT neurons for directions orthogonal to the grating's orientation.
3. The spread of activity across neurons in MT for a low-contrast moving grating will be narrower than for a high-contrast grating (due to signal rectification).
4. Direction opponency and speed are functionally related in MT such that the angular distance between preferred and most opponent directions and preferred speed is negatively correlated.
5. Integration of motion signals in the IOC versus VA directions is related to fast- versus slow-speed tuning. Further, lowering contrast will lead to greater activity in MT's slow-speed tuned neurons.
6. Baseline activity/responsiveness of V1 and/or MT spatiotemporal neurons tuned to low speeds will be higher than those tuned high speeds. While there is some evidence in favor of this idea (Krekelberg et al., 2006; Priebe et al., 2006), we have not seen a systematic assessment of the relationship between spontaneous firing rates and speed preferences.

Just as the methods we used to test MotionNet were inspired by neurophysiological work, future experimental work can be guided by the relationships in the artificial system. For example, to test prediction 4, one could measure direction opponency (Snowden et al., 1991) and speed preference (Mikami et al., 1986) in a population of MT neurons, calculate the angular distance of between the preferred direction and maximum opponency, and then test whether these measures are negatively related. More generally, testing MotionNet on different stimuli and tasks is relatively easy and rapid. This suggests a useful empirical strategy whereby new relationships can be detected and experimental paradigms then optimized to maximize the interpretability of tests. In many cases, it is not feasible to run the same neurophysiological experiment multiple times to best discriminate differing hypotheses. Marrying tests of an artificial system with biological experiments offers an efficient means of uncovering neural architectures.

### Interpreting the role of Bayesian priors

We know human observers systematically misperceive the speed and direction of low-contrast movements. Our findings indicate that this is not due to learning the distribution of environmental motions, as previously believed. Rather, the information structure of natural images means that movement speed and contrast are related. Does this negate previous Bayesian formulations of motion estimation? Our work clearly points to the importance of internalized knowledge of the structure of images and how this affects the visual system. This “prior” is represented by the weight and offset parameters that make MotionNet successful in estimating the speed and direction in most situations. This can be conceptualized within a Bayesian formulation, while noting that previous models are premised on the wrong source of prior knowledge.

As well as proposing the cause of motion illusions in the information structure of natural images, our results suggest a physiological mechanism. We show that bias results from differences in the baseline activity of V1 and MT units (i.e., offset parameters). This could be achieved biologically by differences in the excitability of speed-tuned neurons. Specifically, bias for slow speeds at low contrast may be instantiated through increased excitability of cells tuned to slow speeds. This could be mediated through presynaptic (e.g., differences in excitatory vs inhibitory dendritic connections) and/or postsynaptic mechanisms (e.g., differences in the membrane potential). While there is some evidence for this from previous work (Krekelberg et al., 2006; Priebe et al., 2006), future work is needed to directly test this possibility (e.g., using threshold tracking methods to compare the excitability of speed-tuned neurons in V1 and MT) (Burke et al., 2001).

We previously modeled 3D motion estimation using a “slow world” prior (Welchman et al., 2008). We did not manipulate image contrast but rather exploited differences in the reliability of lateral motion versus motion-in-depth signals. In light of insight from the current paper, it is possible that luminance contrast is a lurking variable in the Welchman et al. (2008) paradigm. Specifically, binocular contrast summation (Meese et al., 2006) would have been stronger for lateral motion trajectories than for motion in depth, with the result that the binocularly higher-contrast lateral movements were perceived as faster. Further experiments are needed to test this possibility.

An alternative account for contrast-dependent misperception hypothesized two speed channels (“slow” vs “fast”) (Thompson, 1982). Under this model, the “fast” channel is silenced at slow speeds at low contrast producing a slower speed estimate. Conversely, at high speeds with low contrast, the “slow” channel is silenced producing a faster speed estimate. This was motivated by evidence that observers overestimate fast speeds when contrast is reduced (Thompson, 1982; Thompson et al., 2006), although this is controversial (Stone and Thompson, 1992; Hawken et al., 1994). We found no evidence for distinct fast versus slow channels; rather, the proportion of MotionNet units as a function of temporal frequency followed a log-normal distribution, similar to macaque V1 and MT neurons (Mikami et al., 1986; Priebe et al., 2006). Moreover, based on the statistical relationship between speed and spatiotemporal contrast, it is not obvious why overestimating speed for low-contrast fast objects would occur. It is possible that very fast motions produce behaviors not captured by the distribution of speeds presented to MotionNet, which may relate to flicker sensitivity rather than speed (Thompson and Stone, 1997).

### Complex motion

Here we trained a shallow network on simple 2D translating image sequences, yet motion is perceived in three spatial dimensions. By training a three-layer network with larger “receptive fields” to make judgments on complex motion, such as optic flow, it may be possible to extract insights that relate to information processing in the medial superior temporal area. It would be informative to observe whether/how altered task demands and additional structure alters the properties of the V1 and MT layers at the single- and population-unit levels. Further, including a third layer releases the second (MT) layer from constrained velocity mapping and allows the units in this layer to develop properties that most effectively support the third (medial superior temporal) layer. Our preliminary work on this indicates that the population-level distribution of direction and speed selectivity among units in the unconstrained MT layer matches those in area MT of biological systems.

## Conclusion

Here we show the key role of image statistics in shaping visual system responses and show how optimizing motion estimation using natural images leads to misperceptions. We make concrete predictions for future experiments, demonstrate the interrelation between movement speed, direction, and spatiotemporal contrast, and show that the human visual system appears to have internalized this information. More generally, the work illustrates how artificial systems approaches can advance understanding of biological computation.
